# Cancer and Its Management 4th Edition

**DOI:** 10.1038/sj.bjc.6602210

**Published:** 2004-11-09

**Authors:** M J Lind

**Affiliations:** 1 The Princess Royal Hospital, UK

This is the fourth edition of a well-known and respected textbook of Oncology by two highly eminent authors. It is always difficult to review textbooks about cancer and its management because inevitably there has to be a compromise between breadth and depth. The authors are to be credited in managing this dilemma. The book manages to cover topics ranging from basic biology of cancer to palliative and terminal care. Each chapter is clearly and concisely written, with a useful summary of the contents of the chapter at its beginning.

Each tumour site is dealt with in a logical manner in describing the aetiology, molecular biology, epidemiology, pathology, diagnosis and treatment. This book will prove invaluable primer in the sublect for a wide range of health care professionals including medical students, specialist nurses, therapy radiographers, specialist registrars in medical and clinical oncology, general practitioners and other physicians and surgeons involved in cancer care.

If there is to be a criticism of this text nit is the criticism that applies to every textbook of Oncology, that it is out of date almost before it is written. However, this is not a major problem as the individual will undoubtedly be stimulated by the excellence of the material within to research their chosen field of interest in more depth.

